# Prevalence of Anxiety and Depression in Prostate Cancer Patients and Their Spouses: An Unaddressed Reality

**DOI:** 10.1155/2020/4393175

**Published:** 2020-01-29

**Authors:** Ernesto Sánchez Sánchez, Antonio Carlos González Baena, Carlos González Cáliz, Fernando Caballero Paredes, José Luis Moyano Calvo, Jesús Castiñeiras Fernández

**Affiliations:** Urology Department, University Hospital “Virgen Macarena”, University of Seville, Seville, Spain

## Abstract

**Objectives:**

To estimate the prevalence of unsuspected anxiety or depression in prostate cancer patients and their spouses, as well as factors involved in its onset. *Materials and Methods*. A prospective study of 184 patients and 137 spouses evaluated in our hospital during 2019 using the Memorial Anxiety Scale for Prostate Cancer (MAX-PC), Hospital Anxiety and Depression Scale (HADS) and Patient Health Questionnaire depression module (PHQ-9). This study provides an internal validity assessment of the scales and their correlation (alpha and rho coefficients; index *r*). The contributions of age, education level, months after diagnosis, pain, prostate-specific antigen (PSA) level, stage of the disease and treatment performed to the positivity of the questionnaires were studied using the Wilcoxon–Mann–Whitney and chi-square tests.

**Results:**

The prevalence of anxiety was 10.9% (MAX-PC) and 28.3% (MAX-PC-PSA). The HADS-A questionnaire indicated pathology in 14.1% of the patients and 16.05% of the spouses. Depression was detected in 7% (HADS-D) and 9.2% (PHQ-9) of patients as well as in 8.8% (HADS-D) and 16.05% (PHQ-9) of their spouses. The greatest concordance between men and women was with the PHQ-9 (Spearman's rho: 0.78; *p* = 0.01). Education level is significantly related to the presence of anxiety and depression, regardless of the questionnaire applied. The probability of detecting pathology in the MAX-PC varied from 6% in patients with elementary education to 23.5% in university students (*p* = 0.04). The greatest differences were detected when applying the PHQ-9 to patients (4% pathological, elementary education vs. 35.3% pathological, university education). Our study confirms the lack of a relationship between rates of anxiety and depression and factors such as PSA level, age of the patient and number of comorbidities.

**Conclusion:**

There is a high prevalence of unsuspected anxiety and depression in patients with prostate cancer and their wives. Education level correlates with such prevalence.

## 1. Introduction

Due to advances in the early diagnosis of prostate cancer (PC) and improvements in treatment in the different phases of the disease, we are witnessing a significant increase in the number of patients who “coexist” with their PC [1]. This increase in survival is not exempt from morbidity, and the functional sequelae of the treatments used have been widely described, including urinary incontinence, erectile dysfunction, intestinal disorders, hot flashes, weakness and fatigue.

Despite the high prevalence of patients and the potential symptomatology of adverse effects, there is a striking lack of research on the nature and prevalence of psychological disorders and psychiatric illness in this population [2–4]. This lack of research is multifactorial, but it may be due in large part to the prioritization of survival outcomes over the quality of life by health professionals involved in the follow-up of these patients. Likewise, there may be a lack of preparation among urologists and oncologists to recognize symptoms of depression among their patients with PC, even if the symptoms are severe. The problem is aggravated because often the wives of patients, who tend to be caregivers, also suffer from anxiety or depression.

Our objectives are to estimate the prevalence of clinically relevant, not previously diagnosed or treated, depressive symptoms in PC patients with good control of their disease and in their spouses, how it is interrelated, and the possible clinical and oncological factors involved in the onset itself.

## 2. Materials, Subjects, and Methods

### 2.1. Patient Selection

Outpatients diagnosed with PC who attend our Urological hospital (January–June 2019) for scheduled monitoring of their disease. Given that the main objective of the study was to detect unknown and/or untreated symptoms, all patients or spouses who were under psychological or pharmacological treatment for anxiety or depression as well as those patients without oncological control of their disease (rising prostate-specific antigen (PSA) level or progression of the disease) or visual analogue scale (VAS) >2 were excluded.

### 2.2. Study Variables

Total scores for questionnaires and pathology percentages, age, months of follow-up since PC diagnosis, stage of the disease (localized, locally advanced, metastatic or castrate-resistance phase-CRPC-), treatment performed (radical prostatectomy (RP), radiotherapy (RT), hormonal treatment (HT), active surveillance (AS) or multimodal treatment), comorbidities, and PSA levels.

### 2.3. Statistical Analysis

Numerical variables are presented as the mean, median, maximum and minimum values, and standard deviation (sd). Categorical variables are presented as the number and percentage. The internal validity of the scales was assessed with Cronbach's alpha and total item correlation (*r*). The correlation between the tests was assessed by determining Spearman's rho coefficient.

The possible influence of the factors studied was performed using Wilcoxon-Mann-Whitney and Pearson's chi-square tests. The statistical package SPSS version 25 was used. A *p*-value of 0.05 or less was considered statistically significant.

### 2.4. Materials and Methods

The clinical baseline information of each patient was collected from the existing data in the clinical history. The tumour stage used was the most recently available.

Patient comorbidities were grouped by system (none, cardiovascular, endocrine, trauma, respiratory, neurological, and other pathologies) and assigned a numerical value based on the number of affected systems.

Pain was assessed according to a VAS scale (0–10).

The following questionnaires validated for the Spanish language were administered:

- Memorial Anxiety Scale for Prostate Cancer (MAX-PC) (patients): The instrument includes 3 subscales: anxiety caused by PSA, fear of disease recurrence and general anxiety. A global score of ≥27 or ≥9 in each subscale was considered clinically significant [5].
- Hospital Anxiety and Depression Scale (HADS) (patients and spouses). The instrument includes 2 subscales (anxiety and depression). Scores ≥11 points were considered pathological [6].
- Patient Health Questionnaire depression module (PHQ-9) (patients and spouses): This instrument was used according to the criteria of the author establishing four groups: Major Depressive Syndrome, Other Depressive Syndrome, Positive Depressive Symptoms and Negative Depressive Symptoms [7].

### 2.5. Ethical Requirements

The research protocol was approved by the ethics committee of our hospital, and all participants gave written informed consent.

## 3. Results

A total of 255 patients were evaluated. Twenty-seven (10.6%) were excluded because they took medication for anxiety or depression, and 14 (5.5%) were excluded because they did not present good oncological control of the disease. Of the 214 remaining patients, a total of 184 (86%) provided informed consent and agreed to be part of the study.

A total of 179 spouses were evaluated, of which 26 (14.5%) were excluded for taking medication. Of the 153 remaining spouses, 137 (89.5%) provided informed consent and completed all the questionnaires.

The population characteristics are provided in Table 1. The median ages of the patients and of their wives were 71 years (49–92) and 68 years (45–87), respectively. The majority had only completed elementary education (53.8%), and only 9.2% had a university degree. The percentage of wives with elementary education was 64.2%. In the majority of cases, patients had undergone RP (53.8%) as primary treatment, 26.6% had initiated HT, 10.9% were included in an AS programme, and 8.7% had been treated with some form of RT. A total of 22.3% had undergone multimodal treatment. The median time from diagnosis to our evaluation disease was 18 months for patients who underwent RP, 12 months for those treated with RT, 23 months for patients who underwent HT and 48 months for those who opted for AS as the first treatment modality.

The mean VAS score was 0.42 (median 0), with 60.9% presenting no pain (VAS 0). None of the scores exceeded 2, which confirms that this population is fundamentally asymptomatic or has very little symptomatology.

The median PSA level was 0.1 (range: 0–88), and all patients were in the response phase to the proposed treatment regardless of the stage of their disease or the treatment performed.

The scores obtained for the questionnaires and their internal validity are presented in Table 2. The median score for the MAX-PC was 17 (11–41 range), and 10.9% of the respondents were considered pathological. The most frequently detected pathological aspect was anxiety caused by PSA (pathological in 28.3% of patients) and, to a lesser extent, general anxiety (10.32%) and fear of recurrence (9.8%).

In the HADS-A questionnaire, 14.1% of patients were considered pathological and 16.05% of the spouses.

Regarding depression in patients, that detected by the HADS-D questionnaire (7.06%) was similar to that detected by the PHQ-9 (9.2%); for spouses; the values were higher: 8.8% were considered depressed according to the HADS-D, and 16.05% according to the PHQ-9.

The internal validity of the questionnaires was very high, with a Cronbach's alpha higher than 0.8 in all cases except for the HADS-D for patients and spouses. The item-total correlation was above the value of 0.35 in almost all cases except for the MAX-PC (0.3) and HADS-D (0.26).

Likewise, the correlation between the different questionnaires according to sex (MAX-PC, HADS-A, HADS-D and PHQ for men and HADS-A, HADS-D and PHQ-9 for the spouses) was excellent, with statistical significance in all possible comparisons (Table 3).

The relationships among PSA variables, age, time since diagnosis, VAS scale and comorbidities are presented in Table 4. The level of statistical significance varied according to the questionnaire applied. PSA values only showed a statistically significant relationship with the global MAX-PC score and with fear of recurrence. Age was only significantly associated with fear of recurrence (MAX-PC). Time since diagnosis was statistically significant for the HADS in both patients and spouses. Level of pain was related to the HADS-D, MAX-PC-A, MAX-PC and PHQ scale for patients. Finally, comorbidities only showed a statistically significant association with the MAX-PC and HADS-D for spouses.

Education level (Table 5) was clearly and consistently related among all the questionnaires. This association is independent of the questionnaire used and was demonstrated equally in spouses. The probability of detecting pathology in the MAX-PC varied from 6% in patients with elementary education to 23.5% in university students. (*p* = 0.04). The greatest differences were detected when applying the PHQ-9 to patients (4% pathological, elementary education vs. 35.3% pathological, university education).

No association was found between the primary treatment used or the stage of the disease and the possibility of presenting a pathological test (Table 6); however, patients who had multimodal treatment had higher levels of anxiety and depression in MAX-PC, HADS-A, and PHQ scales.

## 4. Discussion

The current treatment for PC includes not only an oncological approach to the disease but also a broader vision that includes the global nature of the aspects that influence the health and well-being of the patient and their environment. Anxiety and depression are the most frequent psychological findings in cancer patients [5], and it has been previously reported that patients with PC have higher levels of anxiety and depression than do the general population [8]. These disorders, if not investigated and treated, have a direct negative impact on the overall survival of patients [9].

It is difficult to advise a single instrument for measuring anxiety or depression, as there is no scientific evidence of the superiority of some over others [10]. All the scales used in this study have demonstrated their usefulness, and the percentage of pathological patients indicated was very similar regardless of the method used. In terms of internal consistency, Cronbach's alpha indices were acceptable or good for all scales, always above 0.70 and in the vast majority of cases above 0.80. The high statistical concordance between the results obtained from the questionnaires used in our patients indicates the possibility of using any of the instruments, although the MAX-PC (PSA) is possibly the most robust when determining the anxiety produced by the periodic determination of this marker.

The fundamental findings of the study are the detection of high levels of anxiety and depression in patients with good control of their disease, long after the initial diagnosis (median since diagnosis: 18 months), and who are mostly asymptomatic (median VAS: 0); notably, all those who were in treatment and/or taking medication indicated for anxiety or depression were excluded. To our knowledge, there is no study that has focused on assessing risk in this population, in which, “theoretically”, the risk should be minimal. The median score for the MAX-PC was 17 ± 6.43, which was significantly higher than those previously reported in the literature by Johanes et al. [8] (10.47 ± 4.64), Dale [11] (7.57 ± 7.26) or Rodríguez-Vega et al. [12] (15.7%). The most influential factor is the anxiety produced by the determination of the PSA level (28.3%).

Depression was detected in 7.06% of patients according to the HADS and in 9.2% of patients according to the PHQ-9. A meta-analysis recently published by Watts [13] demonstrated a depression rate of 15–18% in 4000 patients at all stages of PC. Our study confirms that despite the favourable circumstances established in the selection of patients and the known tendency of decreased psychiatric symptoms as the time from initial diagnosis of the disease increases [14], the level of unknown depression is maintained at significantly high levels throughout the course of the disease.

Notably, the levels of anxiety and depression in spouses are slightly higher than those in patients (7.06 vs. 8.8% HADS-D; 9.2 vs. 16.05% PHQ-9), even though the difference did not reach statistical significance (*p* = 0.09). These findings are consistent with those found in the literature [15, 16]. The possible causes of the higher prevalence of anxiety and depression in wives have not been studied; therefore, any interpretation of the data is speculative. Possible reasons include (a) the discordance between communication between couples, assuming that the wife has to openly discuss the problems and feelings of the family unit and the husband wants to minimize the effects and (b) the change in leadership because as the disease progresses, the patient requires more care, which is taken on by his wife, who must become the true support of the family nucleus.

The role of education level in PC anxiety rates has been partially communicated in some studies, with frankly contradictory results. Nelson et al. [17] concluded that patients with elementary level education had higher levels of anxiety regarding PSA levels than did those with a university education. In our study, there was a clear association between education level and rates of anxiety or depression independent of the test used to quantify it. The results were statistically significant and were clearly higher in women. There is no clear explanation for this result. Possibly, patients with higher education levels have greater access to all types of information, greater awareness of the possible negative evolution of the disease, and find it more difficult to reconcile their usual tasks with the inconveniences and deficits they experience living with PC.

Our study confirms the lack of a relationship between rates of anxiety and depression and factors such as PSA level, age of the patient and number of comorbidities. Rates of anxiety and depression were also not related to the initial treatment used or the stage of the disease. These data are consistent with those described by Blank and Bellizzi [18], who concluded that the long-term psychological effects produced by PC depend more on the personality of the patient than on the primary treatment performed or on the side effects that could have occurred. There seems to be a relationship between perceived pain by the patient and the positivity of certain questionnaires. However, these results should be interpreted with caution because 60.9% of the participants did not present any pain (VAS 0). Chabowski et al. [19] evaluated in 2018 the levels of depression, anxiety and pain in patients with lung cancer at risk of malnutrition. It could be another interesting factor, but this item was not analyzed in our population.

## 5. Conclusions

Our results confirm the existence of high rates of anxiety and depression in PC patients and their wives, which are unaddressed by healthcare professionals. In our opinion, this fact justifies the knowledge and periodic use of instruments for their detection.

## 6. Limitations

Despite the consistency of the findings found, we must highlight that the studied population was mostly white, married, with a low level of education and with all health costs covered by the state. Extrapolation of the results to other groups can be difficult because other groups may express their anxiety to the same initial circumstances in a different way.

## Figures and Tables

**Table 1:** General characteristics of the population.

	Mean/median	Range/SD
*Age*		
Patients (*n* = 184)	70.71/71	49–92/8.3
Spouses (*n* = 137)	68.3/68	45–87/7.9

Time since diagnosis (months):	33.3/18	2–142/34.27

PSA (ng/ml)	2.88/0.1	0–88/12.18

*VAS*	0.42/0	0–2/0.54
VAS 0: *n* = 112 (60.9%)		
VAS 1: *n* = 67 (36.4%)		
VAS 2: *n* = 5 (2.7%)		

*Patient education level*	*Spouse education level*	
Elementary: *n* = 99 (53.8%)	Elementary: *n* = 88 (64.2%)	
High school: *n* = 68 (37%)	High school *n* = 37 (27%)	
University: *n* = 17 (9.2%)	University: *n* = 12 (8.8%)	

Comorbidities:	None: 40 (21.7%)	
	Cormob 1: 58 (31.5%)	
	Comorb 2: 64 (34.9%)	
Mean: 1.36	Comorb 3 or more: 22 (11.9%)	
Median: 1		

*Stage* (^∗^)		
Localized *n* = 117 (63.7%)		
L. advanced *n* = 33 (17.9%)		
MTX *n* = 24 (13%)		
CRPC *n* = 10 (5.4%)		

*Primary treatment* (^∗∗^)	*Time since diagnosis (months):*	
AS (*n* = 20; 10.9%)	33.29 (median: 18), range: 2–142	
RP (*n* = 99: 53.8%)	55.9 (median: 48), range: 11–132	
RT (*n* = 16: 8.7%)	30.5 (median: 18), range: 2–142	
HT (*n* = 49: 26.6%)	12.8 (median: 12), range: 2–28	
ST: *n* = 41 (22.3%)	39.8 (median: 23), range: 3–138	

^∗^MTX: metastatic; CRPC: Castrate-resistant prostate cancer. ^∗∗^AS: active surveillance; RP: radical prostatectomy; RT: radiotherapy; HT: hormone therapy; ST: sequential treatment.

**Table 2:** Questionnaire scores and internal consistency.

	Mean/median (range)	SD	Cronbach's alpha	Correlation item/total	Pathological
*MAX-PC patients*	18.57/17 (11–41)	6.43	0.87	0.3 (0.26–0.34)	20/184 (10.9%)
PSA anxiety	7.93/7 (3–17)	2.67	0.83	0.45 (0.3–0.52)	52/184 (28.3%)
Fear of recurrence	4.90/ 5 (0–11)	2.75	0.82	0.43 (0.36–0.5)	18/184 (9.8%)
General anxiety	5.1/5 (2–15)	2.01	0.8	0.4 (0.33–0.46)	19/184 (10.32%)

*HADS patient*					
HADS-A (anxiety)	5.72/5 (1–16)	3.57	0.9	0.48 (0.42–0.54)	26/184 (14.1%)
HADS-D (depression)	4.96/5 (1–12)	2.04	0.71	0.26 (0.20–0.32)	13/184 (7.06%)

*HADS spouse*					
HADS-A (anxiety)	4.63 /4 (0–13)	3.87	0.86	0.48 (0.41–0.56)	22/137 (16.05%)
HADS-D (depression)	3.7/4 (0–13)	3.3	0.79	0.35 (0.28–0.44)	12/137 (8.8%)

*PHQ-9 patient*					
PHQ-9 (*n* = 184)	4.74/6 (0–13)	3.15	0.89	0.601 (0.18–1)	17/184 (9.2%)
Major depressive s.	1/184 (0.5%)				
Other depressive s.	5 (2.7%)				
Depressive s. +	11 (6%)				
Depressive s. −	167 (90.8%)				

*PHQ-9 spouse*					
PHQ-9 (*n* = 137)	4.03/3 (0–10)	3.2	0.88	0.4 (0.37–0.51)	22/137 (16.05%)
Major depressive s.	0 (0%)				
Other depressive s.	6 (4.38%)				
Depressive s. +	16 (11.67%)				
Depressive s. −	115 (83.95%)				

**Table 3:** Correlation among the different questionnaires.

	PHQ-P	HADSA-P	HADSD-P	MAXPS	PHQ-S	HADSA-S	HADSD-S
PHQ-9-P	1.000	.288^∗∗^	.385^∗∗^	.599^∗∗^	.78^∗∗^	.229^∗∗^	.434^∗∗^
HADSA- P	.288^∗∗^	1.000	.241^∗∗^	.472^∗∗^	.463^∗∗^	.86^∗∗^	.580^∗∗^
HADSD-P	.385^∗∗^	.241^∗∗^	1.000	.174^∗^	.313^∗∗^	.025	.103
MAX-PC	.599^∗∗^	.472^∗∗^	.174^∗^	1.000	.555^∗∗^	.070	.358^∗∗^
PHQ-9 -S	.78^∗∗^	.463^∗∗^	.313^∗∗^	.555^∗∗^	1.000	.336^∗∗^	.924^∗∗^
HADSA-S	.229^∗∗^	0.86^∗∗^	.025	.70^∗∗^	.336^∗∗^	1.000	.439^∗∗^
HADSD-S	.434^∗∗^	.580^∗∗^	.103	.358^∗∗^	.924^∗∗^	.439^∗∗^	1.000

Spearman's rho. ^∗∗^The correlation is significant at the 0.01 level (bilateral). ^∗^The correlation is significant at the 0.05 level (bilateral). PHQ-9-P (PHQ-9 patients); PHQ-S (PHQ-9 spouses); HADSA-P (HADS anxiety, patients); HADSA-S (HADS anxiety, spouses); HADSD-P (HADS depression, patients); HADSD-S (HADS depression, spouses).

**Table 4:** Correlation of numerical variables with results from the questionnaires applied.

	PSA	Age	Months d (x)	VAS	Comorb
HADS-A patient	P: 0.1	P: 74	P: 12	P: 0	P: 1
	NP: 0.1	NP: 71	NP: 18.5	NP: 0	NP: 1
	*P* = 0.95	*P* = 0.34	*P* = 0.02	*P* = 0.1^∗^	*P* = 0.49

HADS-D patient	P: 0.15	P: 75	P: 12	P: 1	P: 0
	NP: 0.1	NP: 71	NP: 18	NP: 0	NP: 1
	*P* = 0.4	*P* = 0.39	*P* = 0.09	*P* = 0.06	*P* = 0.65

PHQ-9 patient	P: 0.6	P: 68	P: 15	P: 1	P: 1
	NP: 0.1	NP: 72	NP: 18	NP: 0	NP: 1
	*P* = 0.18	*P* = 0.63	*P* = 0.79	*P* = 0.001	*P* = 0.42

MAX-PC-PSA	P: 0.1	P: 70.5	P: 17	P: 0	P: 1
	NP: 0.1	NP: 72	NP: 18	NP: 0	NP: 0
	*P* = 0.58	*P* = 0.44	*P* = 0.4	*P* = 0.71	*P* = 0.11

MAX-PC-fear recurrence	P: 0.45	P: 77	P: 17	P: 0	P: 1
	NP: 0.1	NP: 71	NP: 18	NP: 0	NP: 1
	*P* = 0.07	*P* = 0.08	*P* = 0.72	*P* = 0.31	*P* = 0.42

MAX-PC-anxiety	P: 0.3	P: 74	P: 19	P: 1	P: 1
	NP: 0.1	NP: 71	NP: 16	NP: 0	NP: 0
	*P* = 0.06	*P* = 0.164	*P* = 0.67	*P* = 0.01	*P* = 0.91

MAX-PC	P: 0.7	P: 73	P: 20	P: 1	P: 2
	NP: 0.1	NP: 71	NP: 18	NP: 0	NP: 1
	*P* = 0.019	*P* = 0.84	*P* = 0.89	*P* = 0.04	*P* = 0.07

HADS-A spouse	P: 0.3	P: 74	P: 26	P: 1	P: 1
	NP: 0.1	NP: 71	NP: 17	NP: 0	NP: 1
	*P* = 0.33	*P* = 0.12	*P* = 0.09	*P* = 0.33	*P* = 0.6

HADS-D spouse	P: 0.6	P: 74	P: 15	P: 1	P: 1
	NP: 0.65	NP: 71	NP: 18	NP: 0	NP: 2
	*P* = 0.32	*P* = 0.32	*P* = 0.02	*P* = 0.13	*P* = 0.001

PHQ-9 spouse	P: 0.37	P: 74	P: 18	P: 1	P: 1
	NP: 0.23	NP: 72	NP: 17	NP: 0	NP: 1
	*P* = 0.39	*P* = 0.71	*P* = 0.45	*P* = 0.03	*P* = 0.42

Mann–Whitney test. P: indicates the median of variables in those cases where the test is pathological, NP: indicates the median of variables in those cases where the test is not pathological. Values are presented as the mean.

**Table 5:** Relation between education level and the questionnaires.

	Elementary education	High school education	University education	Sign (^∗^)
MAX-PC	6/99 (6%)	10/68 (14.7%)	4/17 (23.5%)	*P* = 0.04
HADS anxiety patient	10/99 (10.1%)	12/68 (17.6%)	4/17 (23.5%)	*P* = 0.014
HADS depression patient	4/99 (4%)	6/68 (8.8%)	3/17 (17.6%)	*P* = 0.02
PHQ-9 patient	4/99 (4%)	7/68 (10.3%)	6/17 (35.3%)	*P* ≤ 0.001
HADS anxiety spouse	10/88 (11.4%)	8/37 (21.6%)	4/12 (33.3%)	*P* = 0.01
HADS depression spouse	6/88 (6.8%)	4/37 (10.8%)	2/12 (16.7%)	*P* = 0.05
PHQ-9 spouse	9/88 (10.2%)	6/37 (16.2%)	7/12 (58.3%)	*P* = 0.07

(^∗^) Pearson's chi-square.

**Table 6:** Relationship between primary treatment and stage of the disease with the possibility of the test being pathological.

	HT	RP	RT	AS	*p*(^∗^)	ST	UT	*p* (^∗^)	Local.	L. adv.	Mtx	CRPC	*p* (^∗^)
MAX-PC	5/49 (10.2%)	10/99 (10.1%)	3/16 (18.75%)	2/20 (10%)	0.72	5/41 (12.2%)	15/143 (10.5%)	0.38	11/117 (10.2%)	3/33 (9.09%)	5/24 (20.8%)	1/10 (10%)	0.42
HADS anxiety patient	8/49 (16.3%)	13/99 (13.13%)	3/16 (18.7%)	2/20 (10%)	0.84	5/41 (12.2%)	21/143 (14.7%)	0.69	15/117 (12.8%)	6/33 (18.8%)	4/24 (16.6%)	1/10 (10%)	0.83
HADS depression patient	4/49 (8.16%)	6/99 (6.06%)	2/16 (12.5%)	1/20 (5%)	0.78	3/41 (7.3%)	10/143 (6.9%)	0.94	5/117 (4.2%)	5/33 (15.1%)	2/24 (8.3%)	1/10 (10%)	0.18
PHQ patient	5/49 (10.2%)	8/99 (8.08%)	2/16 (12.5%)	2/20 (10%)	0.93	5/41 (12.2%)	12/143 (8.4%)	0.41	7/117 (5.9%)	4/33 (12.1%)	4/24 (16.6%)	2/10 (20%)	0.19

(^∗^) Pearson's chi-square. ST: sequential treatment; UT: unique treatment; Local.: Localized PC; L. adv.: Locally advanced PC; MTX: metastatic PC; CRPC: castration resistant PC.

## Data Availability

All the data to make the conclusions is included in the article.
